# Supplementary material for: Heat adaptation of phage T7 under an extended genetic code
Source: Virus Evol. 2021 Dec 1;7(2):veab100. doi: 10.1093/ve/veab100 (PMC8923235; doi:10.1093/ve/veab100)
Supplement: veab100_Supp [file veab100_supp.zip › Sequencing_Analysis.zip!/Sequencing_Analysis/S1/evidence/JC_SIDE_2_53.html]

BRESEQ :: Evidence


| New junction evidence | | | | | | | | | | | |
| --- | --- | --- | --- | --- | --- | --- | --- | --- | --- | --- | --- |
|  | | seq id | position | reads (cov) | reads (cov) | score | skew | freq | annotation | gene | product |
| \* | ? | NC\_001604.1 | = 9895 | 4719 (1.120) | 64 (0.020) | 49/592 | 27.8 | 1.3% | intergenic (–/–) | *–/–* | –/– |
|  | ? | NC\_001604.1 | 10215 = | 4867 (1.180) |  |  |  |  | intergenic (–/–) | *–/–* | –/– |
| Rejected: Coverage evenness skew score above cutoff. | | | | | | | | | | | |
| Rejected: Frequency below/above cutoff threshold. | | | | | | | | | | | |

Only 100 of 4969 total aligned reads displayed.  

`AGTGGCAGGGCGCTACGAACAATAAAGGGTACGGGCAGGTGTGGTGCAGCAATACCGGAAAGGTTGTCTACTGTCATCGCGTAATGTCTAATGCTCCGAAAGGTTCTACCGTCCTGCACTCCTGTGATAATCCATTATGTTGTAACCCTGAACACCTATCCATAGGAACTCCAAAAGAGAACTCCACTGACATGGTAAATAAGGGTCGCTCACACAAGGGGTATAAACTTTCAGACGAAGACGTAATGGCAATCATGGAGTCCAGCGAGTCCAATGTATCCTTAGCTCGCACCTATGG‑TGTCTCCCAACAGACTATTTGTGATATACGCAAAGGGAGGCGACATGGCAGGTTACGGCGCTAAAGGAATCCGAAAGGTTGGAGCGTTTCGCTCTGGCCTAGAGGACAAGGTTTCAAAGCAGTTGGAATCAAAAGGTATTAAATTCGAGTATGAAGAGTGGAAAGTGCCTTATGTAATTCCGGCGAGCAATCACACTTACACTCCAGACTTCTTACTTCCAAACGGTATATTCGTTGAGACAAAGGGTCTGTGGGAAAGCGATGATAGAAAGAAGCACTTATTAATTAGGGAGCAGCACCCCG  >  NC_001604.1/9915‑10515`  

                                                                                                                                                                                                                                                                                                             `|`                                                                                                                                                                                                                                                                                                              
   `ggCAGGGCGCTACGAACAATAAAGGGGACGGGCAGGTGTGGTGCAGCAATACCGGAAAGGTTGTCTACTGTCATCGCGTAATGTCTAATGCTCCGAAAGGTTCTACCGTCCTGCACTCCTGCGATAATCCATTATGTTGTAACCCTGAACACCTATCCATAGGAACTCCAAAAGAGAACTCCACTGACATGGTAAATAAGGGTCGCTCACACAA‑GGGTATAAACTTTCAGACGAAGACGTAATGGCAATCATGGAGTACAGCGAGTCCAATGTATCCTTAGCTCGCACCTATGG‑TGTCTcc                                                                                                                                                                                                                                                                                                          <  1:191741/301‑1 (MQ=255)`  
        `agacagaCGAACAATAAAGGGTACGGGCAGGTGTGGTGCAGCAATACCGGAAAGGTTGTCTACTGTCATCGCGTAATGTCTAATGCTCCGAAAGGTTCTACCGTCCTGCACTCCTGTGATAATCCATTATGTTGTAACCCTGAACACCTATCCATAGGAACTCCAAAAGAGAACTCCACTGACATGGTAAATAAGGGTCGCTCACACAAGGGGTATAAACTTTCAGACGAAGACGTAATGGCAATCATGGAGTCCAGCGAGTCCAATGTATCCTTAGCTCGCACCTATGG‑TGTCTCCCAAc                                                                                                                                                                                                                                                                                                      <  1:476945/295‑1 (MQ=255)`  
          `ggcTACGAACAATAAAGGGTACGGGCAGGTGTGGTGCAGCAATACCGGAAAGGTTGTCTACTGTCATCGCGTAATGTCTAATGCTCCGAAAGGTTCTACCGTCCTGCACTCCTGTGATAATCCATTATGTTGTAACCCTGAACACCTATCCATAGGAACTCCAAAAGAGAACTCCACTGACAAGGTAAATAAGGGTCGCTCAAACAAGGGGTATAAACTTTCAGACGAAGACGTAATGGCAATCATGGAGTCCAGCGAGTCCAATGTATCCTTAGCTCGCACCTATGG‑TGTCTCCCAACAg                                                                                                                                                                                                                                                                                                    <  1:508994/300‑1 (MQ=255)`  
                   `cAATAAAGGGTACGGGCAGGTGTGGTGCAGCAATACCGGAAAGGTTGTCTACTGTCATCGCGTAATGTCTAATGCTCCGAAAGGTTCTACCGTCCTGCACTCCTGTGATAATCCATTATGTTGTAACCCTGAACACCTATCCATAGGAACTCCAAAAGAGAACTCCACTGACATGGTAAATAAGGGTCGCTCACACAAGGGGTATAAACTTTCAGACGAAGACGTAATGGCAATCATGGAGTCCAGCGAGTCCAATGTATCCTTAGCTCGCACCTATGGCTGtctcttatacacatctccg                                                                                                                                                                                                                                                                                            >  1:425089/1‑286 (MQ=255)`  
                     `aTAAAGGGTACGGGCAGGTGTGGTGCAGCAATACCGGAAAGGTTGTCTACTGTCATCGAGTAATGTCTAATGCTCCGAAAGGTTCTACCGTCCTGCACTCCTGTGATAATCCATTATGTTGTAACCCTGAACACCTATCCATAGGAACTCCAAAAGAGAACTCCACTGACATGGTAAATAAGGGTCGCTCACACAAGGGGTATAAACTTTCAGACGAAGACGTAATGGCAATCATGGAGTCCAGCGAGTCCAATGTATCCTTAGCTTGCACCTATGG‑GGTCTACCAACAGACTATTTGTGa                                                                                                                                                                                                                                                                                         >  1:520862/1‑301 (MQ=255)`  
                       `cgtgggctcggagatgtgtataagagacagaCCGTAAAGGTTGTCTACTGTCATCGCGTAATGTCTAATGCTCCGAAAGGTTCTACCGTCCTGCACTCCTGTGATAATCCATTATGTTGTAACCCTGAACACCTATCCATAGGAACTCCAAAAGAGAACTCCACTGACATGGTAAATAAGGGTCGCTCACACAAGGGGTATAAACTTTCAGACGAAGACGTAATGGCAATCATGGAGTCCAGCGAGTCCAATGTATCCTTAGCTCGCACCTATGG‑TGTCTCCCAACAGACTATTTGTGata                                                                                                                                                                                                                                                                                       <  1:587288/271‑1 (MQ=255)`  
                                     `ggTGTGGTGCAGCAATACCGGAAAGGTTGTCTACTGTCATCGCGTAATGTCTAATGCTCCGAAAGGTTCTACCGTCCTGCACTCCTGTGATAATCCATTATGTTGTAACCCTGAACACCTATCCATAGGAACTCCAAAAGAGAACTCCACTGACATGGTAAATAAGGGTCGCTCACACAAGGGGTATAAACTTTCAGACGAAGACGTAATGGCAATCATGGATTCCAGCGCGTCCAATGTATCCTTAGCTCGCACCTATGG‑TGTCTCCCAACAGACTATTTGTGCTATACGCCAAGGGAgg                                                                                                                                                                                                                                                                         >  1:158279/1‑301 (MQ=255)`  
                                      `gtgtGGTGCAGCAATACCGGACAGGTTGTCTACTGTCATCGCGTAATGTCTAATGCTCCGAAAGGTTCTACCGTCCTGCACTCCTGTGATAATCCATTATGTTGTAACCCTGAACACCTATCCATAGGAACTCAAAAAGAGACCTCCACTGACATGGTAAATAAGGGTCGCTCACACAAGGGGTATACACTTTCAGACGACGACGTCATGGCACTCCTTGAGTCCAGCCATTCCAATGTCTCCTTAGCTCGCACCTATGG‑TGTTTCCCAACAGCCTATTTACGATATCCGCACAGGGAggg                                                                                                                                                                                                                                                                        >  1:670778/1‑300 (MQ=255)`  
                                           `gtgCAGCAATACCGGAAAGGTTGTCTACTGTCATCGCGTAATGTCTAATGCTCCGAAAGGTTCTACCGTCCTGCACTCCTGTGATAATCCATTATGTTGTAACCCTGAACACCTATCCATAGGAACTCCAAAAGAGAACTCCACTGACATGGTAAATAAGGGTCGCTCACACAAGGGGTATAAACTTTCAGACGAAGACGTAATGGCAATCATGGAGTCCAGCGAGTCCAATGTATCCTTAGCTCGCACCTATGG‑TGTCTCCCAACAGACTATTTGTGatctgtctcttatacacatctca                                                                                                                                                                                                                                                                   >  1:625355/1‑280 (MQ=255)`  
                                                `gcaATACCGGAAAGGTTGTCTACTGTCATCGCGTAATGTCTAATGCTCCGAAAGGTTCTACCGTCCTGCACTCCTGTGATAATCCATTATGTTGTAACCCTGAACACCTATCCATAGGAACTCCAAAAGAGAACTCCACTGACATGGTAAATAAGGGTCGCTCACACAAGGGGTATAAACTTTCAGACGAAGACGTAATGGCAATCATGGAGTCCAGCGAGTCCAATGTATCCTTAGCTCGCACCTATGG‑TGTCTCCCAACAGACTATTTGTGATATACGCCAAGGGAGGCGACCTgtctc                                                                                                                                                                                                                                                              >  1:619967/1‑297 (MQ=255)`  
                                                   `aTACTGGAAAGGTTGTCTACTGTCATCGCGTAATGTCTAATGCTCCGAAAGGTTCTACCGTCCTGCACTCCTGTGATAATCCATTATGTTGTAACCCTGAACACCTATCCATAGGAACTCCAAAAGAGAACTCCACTGACATGGTAAATAAGGGTCGCTCACACAAGGGGTATAAACTTTCAGACGAAGACGTAATGGCAATCATGGAGTCCAGCGAGTCCAATGTATCCTTAGCTCTCACCTATGG‑TGTCTCCCAACAGACTATTTGTGATATACGCAAAgggctgtctcttatacacat                                                                                                                                                                                                                                                           >  1:572045/1‑284 (MQ=255)`  
                                                     `aagggAAAGGTTGTCTACTGTCATCGCGTAATGTCTAATGCTCCGAAAGGTTCTACCGTCCTGCACTCCTGTGATAATCCATTATGTTGTAACCCTGAACACCTATCCATAGGAACTCCAAAAGAGAACTCCACTGACATGGTAAATAAGGGTCGCTCACACAAGGGGTATAAACTTTCAGACGAAGACGTAATGGCAATCATGGAGTCCAGCGAGTCCAATGTATCCTTAGCTCGCACCTATGG‑TGTCTCCCAACAGACTATTTGTGATATACGCAAAGGGAGGCGACATGGCAGGTTAc                                                                                                                                                                                                                                                         <  1:227306/298‑1 (MQ=255)`  
                                                               `ttGTCTACTCTCATCGTGTAATGTCTAATGCTCCGAAAGGTTCTACCGTCCTGCAATCCTGTGATAATCCATTATGTTGTAACCCTGAACACCTATCCATAGGAACTCCAAAAGAGAACTCCAATGACATGGTAAATAAGGGTCGCTCACACAAGGGGTATAAACTTTCAGACGAAGACGTAATGGCAATCATGGAGTCCAGCGAGTCCAATGTATCCTTAGCTCGAACCTATGG‑TGTCTCCCAACAGACTATTTGTGATATACGCAAAGGGAGGCGACATGGCAGGTTACGGCGCTAAAg                                                                                                                                                                                                                                               <  1:648756/301‑1 (MQ=255)`  
                                                                `tGTCTACTGTCATCGCGTAATGTCTAATGCTCCGAAAGGTTCTACCGTCCTGCACTCCTGTGATAATCCATTATGTTGTAACCCTGAACACCTATCCATAGGAACTCCAAAAGAGAACTCCACTGACATGGTAAATAAGGGTCGCTCACACAAGGGGTATAAACTTTCAGACGAAGACGTAATGGCAATCATGGAGTCCAGCGAGTCCAATGTATCCTTAGCTCGCACCTATGG‑TGTCTCCCAACAGACTATTTGTGATATACGCAAAGGGAGGCGACATGGCAGGTTACGGCGCTAAAgg                                                                                                                                                                                                                                              <  1:133772/301‑1 (MQ=255)`  
                                                                               `cgTAATGTCTAATGCTCCGAAAGGTTCTACCGTCCTGCACTCCTGTGATAATCCATTATGTTGTAACCCTGAACACCTATCCATAGGAACTCCAAAAGAGAACTCCACTGACATGGTAAATAAGGGTCGCTCACACAAGGGGTATAAACTTTCAGACGAAGACGTAATGGCAATCATGGAGTCCAGCGAGTCCAATGTATCCTTAGCTCGCACCTATGG‑TGTCTCCCAACAGACTATTTGTGATATACGCAAAGGGAGGCGACATGGCAGGTTACGGCGCtgtctcttatacacatctccc                                                                                                                                                                                                                               >  1:21350/1‑281 (MQ=255)`  
                                                                                    `tGTCTAATGCTCCGAAAGGTTCTACCGTCCTGCACTCCTGTGATAATCCATTATGTTGTAACCCTGAACACCTATCCATAGGAACTCCAAAAGAGAACTCCACTGACATGGTAAATAAGGGTCGCTCACACAAGGGGTATAAACTTTCAGACGAAGACGTAATGGCAATCATGGAGTCCAGCGAGTCCAATGTATCCTTAGCTCGCACCTATGG‑TGTCTCCCAACAGACTATTTGTGATATACGCAAAGGGAGGCGACATGGCAGGTTACGGCGCTAAAGGAATCCGAAAGGttctgtctc                                                                                                                                                                                                                          >  1:33103/1‑294 (MQ=255)`  
                                                                                            `gCTCCGAAAGGTTCTACCGTCCTGCACTCCTGTGATAATCCATTATGTTGTAACCCTGAACACCTATCCATAGGAACTCCAAAAGAGAACTCCACTGACATGGTAAATAAGGGTCGCTCACACAAGGGGTATAAACTTTCAGACGAAGACGTAATGTCAATCATGGAGTCCAGCGAGTCCAATGTATCCTTAGCTCGCACCTATGG‑TTTCTCCCAACTGACTATTTGTAATATACGCAAAGGGAGTCGACTTGGCAGGTTACGGCGCTACAGGAATCCAAAAGGTTGGAGCGTTTCGctct                                                                                                                                                                                                                  >  1:282938/1‑301 (MQ=255)`  
                                                                                             `cTCCGCAAGGTTCTACCGTCCTGCACTCCTGTGATAATCCATTATGTTGTAACCCTGAACACCTATCCATAGGAACTCCAAAAGAGAACTCCACTGACATGGTAAATAAGGGTCGCTCACACAAGGGGTCTAAACTTTCAGACGAAGACGTAATGGCAATCATGGAGTCAAGCGAGTCCAATGTATCCTTCGCTCGCACCTATGG‑TGTCTCCCAACAGACTATTTGTGATATACGCCAAGCGAGGCGACATGGCAGGTTACGGCGCTAAAGGAATCCGAAAGGTTGGAGCGTTTCGCTCTg                                                                                                                                                                                                                 >  1:185179/1‑301 (MQ=255)`  
                                                                                                     `ggTTCTACCGTCCTTCACTCCTGTGATAATCCATTATGTTGTAACCCTGAACACCTATCCATAGGAACTCCAAAAGAGAACTCCACTGACATGGTAAATAAGGGTCGCTCACACAAGGGGTATAAACTTTCAGACGAAGACGTAATGGCAATCATGGAGTCCAGCGAGTCCAATGTATCCTTAGCTCGCACCTATGG‑TGTCTCCCAACAGACTATTTGTGATATACGCAAAGGGAGGCGACATGGCAGGTTACGGCGCTAAAGGAATCCGAAAGGTTGGAGCGTTTCGCTCTGGCCTagag                                                                                                                                                                                                         <  1:221686/301‑1 (MQ=255)`  
                                                                                                      `gTTCTACCGTCCTGCACTCCTGTGATAATCCATTATGTTGTAACCCTGAACACCTATCCATAGGAACTCCAAAAGAGAACTCCACTGACATGGTAAATAAGGGTCGCTCACACAAGGGGTATAAACTTTCAGACGCAGACGTAATGGCAATCATGGAGTCCAGCGAGTCCAATGTATCCTTAGCTCGCACCTATGG‑TGTCTCCCAACAGACTATTTGTGATATACGCAAAGGGCGGCGACATGCCAGGTTACGGCGCTAAAGGAATCCGAAAGGTTGGAGCGTTTCGCTCTGGCCTAGAgg                                                                                                                                                                                                        >  1:354056/1‑301 (MQ=255)`  
                                                                                                         `cTACCGTCCTGCACTCCTGTGATAATCCATTATGTTGTAACCCTGAACACCTATCCATAGGAACTCCAAAAGAGAACTCCACTGACATGGTAAATAAGGGTCGCTCACACAAGGGGTATAAACTTTCAGACGAAGACGTAATGGCAATCATGGAGTCCAGCGAGTCCAATGTATCCTTAGCTCGCACCTATGG‑TGTCTCCCAACAGACTATTTGTGATATACGCAAAGGGAGGCGACATGGCAGGTTACGGCGCTAAAGGAATCCGAAAGGTTGGAGCGTTTCGCTCTGGCCTAGAGGaaa                                                                                                                                                                                                     >  1:113131/1‑299 (MQ=255)`  
                                                                                                                   `gCACTCCTGTGATAATCCATTATGTTGTAACCCTGAACACCTATCCATAGGAACTCCAAAAGAGAACTCCACTGACATGGTAAATAAGGGTCGCTCACACAAGGGGTATAAACTTTCAGACGAAGACGTAATGGCAATCATGGAGTCCAGCGAGTCCAATGTATCCTTAGCTCGCACCTATGG‑TGTCTCCCAACAGACTATTTGTGATATACGCAAAGGGAGGCGACATGGCAGGTTACGGCGCTAAAGGAATCCGAAAGGTTGGAGCGTTTCGCTCTGGCCTAGGGGACAAGGTTTCaaa                                                                                                                                                                                           >  1:358751/1‑301 (MQ=255)`  
                                                                                                                   `gCACTCCTGTGATAATCCATTATGTTGTAACCCTGAACACCTATCCATAGGAACTCCAAAAGAGAACTCCACTGACATGGTAAATAAGGGTCGCTCACACAAGGGGTATAAACTTTCAGACGAAGACGTAATGGCAATCATGGAGTCCAGCGAGTCCAATGTATCCTTAGCTCGCACCTATGG‑TGTCTCACAACAGACTATTTGTGATATACGCACAGGGAGGCGACATGGCAGGTTACGGCGCTAAAGGAATCCGAAAGGTTGGAGCGTTTCGCTCTGGCCTAGTGGACAAGCTTTcctg                                                                                                                                                                                           >  1:483637/1‑298 (MQ=255)`  
                                                                                                                     `aCTCCGGTGATAAACCATTATGGTGTAACCCTGAACACCTATCCATAGGAACTCCAAAAGAGAACTCCACTGACATGGTAAATAAGGGTCGCTCACACAAGGGGTATAAACTTTCAGACGAAGACGTAATGGCAATCATGGAGTCCAGCGAGTCCAATGTATCCTTAGCTCGCACCTATGG‑TGTCTCCCAACAGACTATTTGTGATATACGCAAAGGGAGGCGACATGGCAGGTTACGGCGCTAAAGGAATCCGAAAGGTTGGAGCGTTTCGCTCTGGCCTAGAGGACAAGGTTTCAAAGc                                                                                                                                                                                         <  1:88886/301‑1 (MQ=255)`  
                                                                                                                      `cTCCTGTGCTAATCCATTATGTTGTAACCCTGAACACCTATCCATAGGAACTCCAAAAGAGAACTCCACTGACATGGTAAATAAGGGTCGCTCACACAAGGGGTATAAACTTTCAGACGAAGACGTAATGGCAATCATGGAGTCCAGCGAGTCCAATGTATCCTTAGCTCGCACCTATGG‑TGTCTCCCAACAGACTATTTGTGATATACGCAAAGGGAGGCGACATGGCAGGTAACGGCGCTAAAGGAATCCGAAAGGTTGGAGCGTTTCGCTCTGGCCTAGAGGACAAGGTTTCAAAGCa                                                                                                                                                                                        >  1:246946/1‑301 (MQ=255)`  
                                                                                                                      `cTCCTGTGATAATCCATTATGTTGTAACCCTGAACACCTATCCATAGGAACTCCAAAAGAGAACTCCACTGACTTGGTAAATAAGGGTCGCTCACACAAGGGGTATAAACTTTCAGACGAAGACGTAATGGCAATCATGGAGTCCAGCGAGTCCAATGTATCCTTAGCCCGCACCTATGG‑TGTCTCCCAACAGACTATTTGTGATATACGCAAAGGGAGGCGACATGGCAGGTTACGGCGCTAAAGGAATCCGAAAGGTTCGAGCGTTTCGCTCTGGccctgtcccttatacacacctccg                                                                                                                                                                                        >  1:437002/1‑279 (MQ=255)`  
                                                                                                                          `aagagacagccATTATGTTGGAACCCGTAACACCTATCCATAGGAACTCCAAAAGAGAACTCCACTGACATGGTAAATAAGGGTCGCTCACACAAGGGGTATAAACTTTCAGACGAAGACGTAATGGCAATCATGGAGTCCAGCGAGTCCAATGTATCCTTAGCTCGCACCTATGG‑TGTCTCCCAACAGACTATTTGTGATATACGCAAAGGGAGGCGACATGGCAGGTTACGGCGCTAAAGGAATCCGAAAGGTTGGAGCGTTTCGCTCTGGCCTAGAGGACAAGGTTTCAAAGCAGTTg                                                                                                                                                                                    <  1:93589/292‑1 (MQ=255)`  
                                                                                                                           `gtgATAATCCATTATGTTGTAACCCTGAACACCTATCCATAGGAACTCCAAAAGAGAACTCCACTGACATGGTAAATAAGGGTCGCTCACACAAGGGGTATAAACTTTCAGACGAAGACGTAATGGCAATCATGGAGTCCAGCGAGTCCAATGTATCCTTAGCTCGCACCTATGG‑TGTCTCCCAACAGACTATTTGTGATATACGCATAGGGAGGCGACATGGCAGGTTACGGCGCTAAAGGAATCCGAAAGGATGGAGCGGTTCGCTCTGGCCTAGAGGACAAGGTTCCAAAGCAGTTgg                                                                                                                                                                                   >  1:375055/1‑301 (MQ=255)`  
                                                                                                                           `gtgATAATCCATTATGTTGTAACCCTGAACACCTATCCATAGGAACTCCAAAAGAGAACTCCACTGACATGGTAAATAAGGGTCGCTCACACAAGGGGTATAAACTTTCAGACGAAGACGTAATGGCAATCATGGAGTCCAGCGAGTCCAATGTATCCTTAGCTCGCACCTATGG‑TGTCTCCCAACAGACTATTTGTGATATACGCAAAGGGAGGCGACATGGCAGGTTACGGCGCTAAAGGAATCCGAAAGGTTGGTGCGTTTCGCTCTGGCCTAGAGGACAAGGTTTCAAAGCAGtttg                                                                                                                                                                                   >  1:259827/1‑299 (MQ=255)`  
                                                                                                                           `gtgATAATCCATTATGTTGTAACCCTGAACACCTATCCATAGGAACTCCAAAAGAGAACTCCACTGACATGGTAAATAAGGGTCGCTCACACAAGGGGTATAAACTTTCAGACGAAGACGTAATGGCAATCATGGAGTCCAGCGAGTCAAATGTATCCTTAGCTCGCACCTATGG‑TGTCTCCCAACAGACTATTTGTGATATACGCAAAGGGAGGCGACATGGCAGGTTACGGCGCTAAAGGAATCCGAAAGGTTGGAGCGTTTCGCTCTGGCCTAGAGGACAAGGTTTCAAAGCAGTTgg                                                                                                                                                                                   >  1:501788/1‑301 (MQ=255)`  
                                                                                                                                   `ccATTATGTTGTAACCCTGAACACCTATCCATAGGAACTCCAAAAGAGAACTCCACTGACATGGTAAATAAGGGTCGCTCACACAAGGGGTATAAACTTTCAGACGAAGACGTAATGGCAATCATGGAGTCCAGCGAGTCCAATGTATCCTTAGCTCGCACCTATGG‑TGTCTCCCAACAGACTATTTGTGATATACGCAAAGGGAGGCGACATGGCAGGTTACGGCGCTAAAGGAATCCGAAAGGTTGGAGCGTTTCGCTCTGGCCTAGAGGACAAggctgtctcttatacacatctccga                                                                                                                                                                           >  1:266744/1‑278 (MQ=255)`  
                                                                                                                                        `aTGTTGTAACCCTGAACACCTATCCATAGGAACTACAAAAGAGAACTCCACTGACATGGTAAATAAGGGTCGATCACACAAGGGGTATAAACTTTCAGACGAAGACGTAATGGCAATCATGGAGTCAAGCGAGTCCAATGTATCCTTAGCTCGCACCTATGG‑TGTCTCCCAACAGACTATTTGTGATATACGAAAAGGGAGGCGACATGGCAGGTTACGGCGCTAAAGGAATCCGACAGGTTGGAGCGTTTCGCTCTGGCCTAGAGGACAAGGTTTCAAAGCAGTTGGAATACAACGGTAt                                                                                                                                                                      >  1:311702/1‑301 (MQ=255)`  
                                                                                                                                          `gttgtAACCCTGAACACCTATCCATAGGAACTCCAAAAGAGAACTCCACTGACATGGTAAATAAGGGTCGCTCACACAAGGGGTATAAACTTTCAGACGAAGACGTAATGGCAATCATGGAGTCCAGCGAGTCCAATGTATCCTTAGCTCGCACCTATGG‑TGTCTCCCAACAGACTATTTGTGATATACGCAAAGGGAGGCGACATGGCAGGTTACGGCGCTAAAGGAATCCGAAAGGTTGGAGCGTTTCGCTCTGGCCTAGAGGACAAGGTTTCAAAGCAGTTGGAATCAAAAGGTATTa                                                                                                                                                                    >  1:164906/1‑301 (MQ=255)`  
                                                                                                                                           `ttgtAACCCTGAACACCTATCCATAGGAACTCCAAAAGAGAACTCCACTGACATGGTAAATAAGGGTCGCTCACACAAGGGGTATAAACTTTCAGACGAAGACGTAATGGCAATCATGGAGTCCAGCGAGTCCAATGTATCCTTAGCTCGCACCTATGG‑TGTCTCCCAACAGACTATTTGTGATATACGCAAAGGGAGGCGACATGGCAGGTTACGGCGCTAAAGGAATCCGAAAGGTTGGAGCGTTTCGCTCTGGCCTAGAGGACAAGGTTTCAAAGCAGTTGGAATCAAAAGGTATTaa                                                                                                                                                                   >  1:458433/1‑301 (MQ=255)`  
                                                                                                                                              `tAACCCTGAACACCTATCCATAGTAACTCCAAAAGAGAACTCCACTGACATGGTAAATAAGGGTCGCTCACACAAGGGGTATAAACTTTCAGACGAAGACGTAATGGCAATCATGGAGTCCAGCGAGTCCAATGTATCCTTAGCTCGCACCTATGG‑TGTCTCCCAACAGACTATTTGTGATATACGCAAAGGGAGGCGACATGGCAGGTTACGGCGCTAAAGGAATCCGAAAGGTTGGAGCGTTTCGCTCTGGCCTAGAGGACAAGGTTTCAAAGCAGTTGGAATCAAAAGGTATTAAAtt                                                                                                                                                                <  1:653481/301‑1 (MQ=255)`  
                                                                                                                                                `aCCCTGAACACCTATCCATAGGAACTCCAAAAGAGAACTCCACTGACATGGTAAATAAGGGTCGCTCACACAAGGGGTATAAACTTTCAGACGAAGACGTAATGGCAATCATGGAGTCCAGCGAGTCCAATGTATCCTTAGCTCGCACCTATGG‑TGTCTCCCAACAGACTATTTGTGATATACGCAAAGGGAGGCGACATGGCAGGTTACGGCGCTAAAGGCATCCGAAAGGTTGGAGCGTTTCGCTCTGGCCTAGAGGACAAGGTTTCAAAGCAGTTGGAATCAAACGGTATTAAATTCg                                                                                                                                                              >  1:387924/1‑301 (MQ=255)`  
                                                                                                                                                 `cccTGAACACCTATCCATAGGAACTCCAAAAGAGAACTCCACTGACATGGTAAATAAGGGTCGCTCACACAAGGGGTATAAACTTTCAGACGATGACGTAATGGCAATCATGGAGTCCAGCGAGTCCAATGTATCCTTAGCTCGCACCTATGG‑TGTCTCCCAACAGACTATTTGTGATATACGCAAAGGGAGGCGACATGGCAGGTTACGGCGCTAAAGGAATCCGAAAGGTTGGAGCGTTTCGCTCTGGCCTAGAGGACAAGGGTTCAAAGGAGGTGGAATCAAAAGGGATTaaactgta                                                                                                                                                             >  1:71354/1‑296 (MQ=255)`  
                                                                                                                                                          `ccTATCCATAGGAACTCCAAAAGAGAACTCCACTGACATGGTAAATAAGGGTCGCTCACACAAGGGGTATAAACTTTCAGACGAAGACGTAATGGCAATCATGGAGTCCAGCGAGTCCAATGTATCCTTAGCTCGCACCTATGG‑TGTCTCCCAACAGACTATTTGTGATATACGCAAAGGGAGGCGACATGGAAGGTTACGGCGCTAAAGGAATCCGAAAGGTTGGAGCGTTTCGCTCTGGCCTAGAGGACAAGATTTCAAAGCAGTTGGAATCAAACGGTATTAAATTCGAGTATGAaga                                                                                                                                                    >  1:407546/1‑301 (MQ=255)`  
                                                                                                                                                          `ccTATCCATAGGAACTCCAAAAGAGAACTCCACTGACATGGTAAATAAGGGTCGCTCACACAAGGGGTATAAACTTTCAGACGAAGACGTAATGGCAATCATGGAGTCCAGCGAGTCCAATGTATCATTAGCTCGCACCTATGG‑TGTCTCCCAACAGACTATTTGTGATATACGCAAAGGGAGGCGACATGGCAGGTTACGGCGCTAAAGGAATCCGAAAGGTTGGAGCGTTTCGCTCTGGCCTAGAGGACAAGGTTTCAAAGCAGTTGGAATCAAAAGGTATTAAATTCGAGTATGAaga                                                                                                                                                    >  1:305624/1‑301 (MQ=255)`  
                                                                                                                                                             `aTCCATAGGAACTCCAAAAGAGAACTCCACTTACATGGTAAATAAGGGTCGCTCACACAAGGGGTATAAACTTTCAGACGACGACGTAATGGCAATCATGGAGTCCAGCGAGTCCAATGTATCCTTAGCTCGCACCTATGG‑TGTCTCCCAACAGACTATTTGTGATATACGCAAAGGGAGGCGACATGGCAGGTTACGGCGCTAAAGGAATCCGAAAGGTTGGAGCGTTTCGCTCTGGCCTAGAGGACAAGGTTTCAAAGCAGTTGGAATCAAACGGTATTAAATTCGAGTATGAAGAGtc                                                                                                                                                 >  1:514805/1‑300 (MQ=255)`  
                                                                                                                                                             `aTCCATAGGAACTCCAAAAGAGAACTCCACTGACATGGTAAATAAGGGTCGCTCACACAAGGGGTATAAACTTTCAGACGAAGACGTAATGGCAATCATGGAGTCCAGCGAGTCCAATGTATCCTTAGCTCGCACCTATGG‑TGTCTCCCAACAGACTATTTGTGATATACGCAAAGGGAGGCGACATGGCAGGTTACGGCGCTAAAGGAATCCGAAAGGTTGGAGCGTTTCGCTCTGGCCTAGAGGACAAGGTTTCAAAGCAGTTGGAATCAAAAGGTATTAAATTCGAGTATGAAGAGTg                                                                                                                                                 >  1:2335/1‑301 (MQ=255)`  
                                                                                                                                                              `tCCATAGGAACTCCAAAAGAGAACTCCACTGACATGGTAAATAAGGGTCGCTCACACAAGGGGTATAAACTTTCAGACGAAGACGTAATGGCAATCATGGAGTCCAGCGAGTCCAATGTATCCTTAGCTCGCACCTATGG‑TGTCTCCCAACAGACTATTTGTGATATACGCAAAGGGAGGCGACATGGCAGGTTACGGCGCTAAAGGAATCCGAAAGGTTGGAGCGTTTCGCTCTGGCCTAGAGGACAAGGTTTCAAAGCAGTTGGAATCAAAAGGTATTAAATTCGAGTATGAAGAGTgg                                                                                                                                                >  1:594615/1‑301 (MQ=255)`  
                                                                                                                                                                `gtataagagacagaaaGAGGACCCCACTGACATGGTAAATAAGGGTCGCACACCCAAGGGGTATAAACTTTCAGACGAAGACGGAATGGCAATCATGGAGTCCAGCGAGTCCAATGTATCCTTAGCTCGCACCTATGG‑TGTCTCCCAACAGACTATTTGTGATATACGCAAAGGGAGGCGACATGGCAGGTTACGGCGCTAAAGGAATCCGAAAGGTTGGAGCGTTTCGCTCTGGCCTAGAGGACAAGGTTTCAAAGCAGTTGGAATCAAAAGGTATTAAATTCGAGTATGAAGAGTGGaa                                                                                                                                              <  1:34132/288‑1 (MQ=255)`  
                                                                                                                                                                 `aTAGGAACTCCAAAAGATAACTCCACTGACATGGTAAATAAGGGTCGCTCACACAAGGGGTATAAACTTTCAGACGAAGACGTAATGGCAATCATGGAGTCCAGCGAGTCCAATGTATCCTTAGCTCGCACCTATGG‑TGTCTCCCAACAGACTATTTGTGATATACGCAAAGGGAGGCGACATGGCAGGTTACGGCGCTAAAGGAATCCGAAAGGTTGGAGCGTTTCGCTCTGGCCTAGAGGACAAGGTTTCAAAGCAGTTGGAATCAAAAGGTATTAAATTCGAGTATGAAGAGTGGaaa                                                                                                                                             >  1:348306/1‑301 (MQ=255)`  
                                                                                                                                                                  `aaGGAACTCCAAAAGAGAACTCCACTGACATGGTAAATAAGGGTCGCTCACACAAGGGGTATAAACTTTCAGACGAAGACGTAATGGCAATCATGGAGTCCAGCGAGTCCAATGTATCCTTAGCTCGCACCTATGG‑TGTCTCCCAACAGACTATTTGTGATATACGCAAAGGGAGGCGACATGGCAGGTTACGGCGCTAAAGGAATCCGAAAGGTTGGAGCGTTTCGCTCTGGCCTAGAGGACAAGGTTTCAAAGCAGTTGGAATCAAAAGGTATTAAATTCGAGTATGAAGAGTGGAAAg                                                                                                                                            <  1:488885/300‑1 (MQ=255)`  
                                                                                                                                                                     `cagcTCCAAAAGAGAACTCCACTGACATGGTAAATAAGGGTCGCTCACACAAGGGGTATAAACTTTCAGACGAAGACGTAATGGCAATCATGGAGTCCAGCGAGTCCAATGTATCCTTAGCTCGCACCTATGG‑TGTCTCCCAACAGACTATTTGTGATATACGCAAAGGGAGGCGACATGGCAGGTTACGGCGCTAAAGGAATCCGAAAGGTTGGAGCGTTTCGCTCTGGCCTAGAGGACAAGGTTTCAAAGCAGTTGGAATCAAAAGGTATTAAATTCGAGTATGAAGAGTGGAAAGTGc                                                                                                                                         <  1:453040/298‑1 (MQ=255)`  
                                                                                                                                                                          `ccAAAAGAGAACTCCACTGACATGGTAAATAAGGGTCGCTCACACAAGGGGTATAAACTTTCAGACGAAGACGTAATGGCAATCATGGAGTCCAGCGAGTCCAATGTATCCTTAGCTCGCACCTATGG‑TGTCTCCCAACAGACTATTTGTGATATACGCAAAGGGAGACGACATGGCAGGTTACGGCGCTAAAGGAATCCGAAAGGTTGGAGCGTTTCGCTCTGGCCTAGAGGACAAGGTTTCAAAGCAGTTGGAATCAAAAGGTATTAAATTCGAGTATGAagagctgtctcttatacac                                                                                                                                    >  1:318399/1‑286 (MQ=255)`  
                                                                                                                                                                           `cAAAAGAGAACTCCACTGACATGGTAAATAAGGGTCGCTCACACAAGGGGTATAAACTTTCAGACGAAGACGTAATGGCAATCATGGAGTCCAGCGAGTCCAATGTATCCTTAGCTCGCACCTATGG‑TGTCTCCCAACAGACTATTTGTGATATACGCAAAGGGAGGCGACATGGCAGGTTACGGCGCTAAAGGAATCCGAAAGGTTGGAGCGTTTCGCTCTGGCCTAGAGGACAAGGTTTCAAAGCAGTTGGAATAAAAAGGTATTAAATTCGAGTATGAAGAGTGGAAAGTGCCTTATg                                                                                                                                   <  1:138701/301‑1 (MQ=255)`  
                                                                                                                                                                                 `agaACTCCACTGACATGGTAAATAAGGGTCGCTCACACAAGGGGTATAAACTTTCAGACGAAGACGTAATGGCAATCATGGAGTCCAGCGAGTCCAATGTATCCTTAGCTCGCACCTATGG‑TGTCTCCCAACAGACTATTTGTGATATACGCAAAGGGAGGCGACATGGCAGGTTACGGCGCTAAAGGAATCCGAAAGGTTGGAGCGTTTCGCTCTGGCCTAGAGGACAAGGTTTCAAAGCAGTTGGAATCAAAAGGTATTAAATTCGAGTATGAAGAGTGGAAAGTGCCTTATGTAATTc                                                                                                                             <  1:100093/301‑1 (MQ=255)`  
                                                                                                                                                                                  `gaACTCCACTGACATGGTAAATAAGGGTCGCTCACACAAGGGGTATAAACTTTCAGACGAAGACGTAATGGCAATCATGGAGTCCAGCGAGTCCAATGTATCCTTAGCTCGCACCTATGG‑TGTCTCCCAACAGACTATTTGTGATATACGCAAAGGGAGGCGACATGGCAGGTTACGGCGCTAAAGGAATCCGAAAGGTTGGAGCGTTTCGCTCTGGCCTAGAGGACAAGGTTTCAAAGCAGTTGGAATCAAAAGGTATTAAATTCGAGTATGTAGAGTGGAAAGTGCCTTATGTAATTcc                                                                                                                            >  1:495509/1‑301 (MQ=255)`  
                                                                                                                                                                                  `gaACTCCACTGACATGGTAAATAAGGGTCGCTCACACAAGGGGTATAAACTTTCAGACGAAGACGTAATGGCAATCATGGAGTCCAGCGAGTCCAATGTATCCTTAGCTCGCACCTATGG‑TGTCTCCCAACAGACTATTTGTGATATACGCAAAGGGAGGCGACATGGCAGGTTACGGCGCTAAAGGAATCCGAAAGGTTGGAGCGTTTCGCTCTGGCCTAGAGGACAAGGTTTCAAAGCAGTTGGAATCAAAAGGTATTAAATTCGAGTATGAAGAGTGGAAAGTGCCTTATGTAATTcc                                                                                                                            >  1:144663/1‑301 (MQ=255)`  
                                                                                                                                                                                     `cTCCACTGACATGGTAAATAAGGGTCGCTCACACAAGGGGTATAAACTTTCAGACGAAGACGTAATGGCAATCATGGAGTCCAGCGAGTCCAATGTATCCTTAGCTCGCACCTATGG‑TGTCTCCCAACAGACTATTTGTGATATACGCAAATGGAGGCGACATGGCAGGTTACGGCGCTAAAGGAATCCGAAAGGTTGGAGCGTTTCGCTCTGGCCTAGAGGACAAGGTTTCAAAGCAGTTGGAATCAAAAGGTATTAAATTCGAGTATGAAGAGTGGAAAGTGCCTTATGTAATTCCGGc                                                                                                                         >  1:539235/1‑301 (MQ=255)`  
                                                                                                                                                                                            `gACATGGTAAATAAGGGTCGCTCACACAAGGGGTATAAACTTTCAGACGAAGACGTAATGGCAATCATGGAGTCCAGCGAGTCCAATGTATCCTTAGCTCGCACCTATGG‑TGTCTCCCAACAGACTATTTGTGATATACGCAAAGGGAGGCGACATGGCAGGTTACGGCGCTAAAGGAATCCGAAAGGTTGGAGCGTTTCGCTCTGGCCTTGAGGACAAGGTTTCAAAGAAGTTGGAATCAAAAGGTATTAAATTCGAGTATGAAGCGTGGAAACTGTCtcttatacccatctccgagccc                                                                                                                  >  1:567041/1‑280 (MQ=255)`  
                                                                                                                                                                                            `gACATGGTAAATAAGGGTCGCTCACACAAGGGGTATAAACTTTCAGACGAAGACGTAATGGCAATCATGGAGTCCAGCGAGTCCAATGTATCCTTAGCTCGCACCTATGG‑TGTCTCCCAACAGACTATTTGTGATATACGCAAAGGGAGGCGACATGGCAGGTTACGGCGCTAAAGGAATCCGAAAGGTTGGAGCGTTTCGCTCTGGCCTAGAGGACAAGGTTTCAAAGCAGTTGGAATCAAAAGGTATTAAATTCGAGTATGAAGAGTGGAAAGTGCCTTATGTAATTCCGGCGAGCAAt                                                                                                                  >  1:197252/1‑301 (MQ=255)`  
                                                                                                                                                                                                 `ggTAAATAAGGGTCGCTCACACAAGGGGTATAAACTTTCAGACGAAGACGTAATGGCAATCATGGAGTCAAGCGAGTCCAATGTATCCTTAGCTCGCACCTATGG‑TGTCTCCCAACAGACTATTTGTGATATACGCAAAGGGAGGCGACATGGCAGGTTACGGCGCTAAAGGAATCCGAAAGGTTGGAGCGTTTCGCTCTGGCCTAGAGGACAAGGTTTCAAAGCAGTTGGAATCAAAAGGTATTAAATTCGAGTATGAAGAGTGGAAAGTGCCTTATGTAATTCCGGCGAGCAATCacac                                                                                                             <  1:413129/301‑1 (MQ=255)`  
                                                                                                                                                                                                 `ggTAAATAAGGGTCGATCAAACAAGGGGTATAAACTTTCAGACTAAGACGTAATGGCAATCATGGAGGCCAGCGAGTCCAATGTATCCTTAGCTCGCACCTATGG‑TGTCTCCCAACATACTATTTGTGATATACGCAAAGGGAGGCGACATGGCAGGTTACGGCGCTAAAGGAATCCGAAAGGTTGGAGCGTTTCGCTCTGGCCTAGAGGACAAGGTTTCAAAGCAGTTGGAATCAAAAGGTATTAAATTCGAGTATGAAGAGTGGAAAGTGCCTTATGTAATTCCGGCGAGCAATCacac                                                                                                             <  1:578960/301‑1 (MQ=255)`  
                                                                                                                                                                                                   `tAAAGAAGGGTCGCACAAACAAGGGGTTATAACTGTCAGAGGAAGACGTAAAGGACATCATGGATGCCAGCGAGTCCAATGTATCCTTAGCTCGCACCTATGG‑TGTCTCCCAAACGACTAGTTGTGATATACGCAAAGGGAGGTGACATGGCAGGTTACGGCGCTAAAGGAATCCGAAAGGTTGGAGGGTTTCGCTCTCGCCTAGAGGACAAGGTTTCAAAGCATGTGGAATCAAAAGGTATTAAATTCGAGTATGAAGAGTGGAAAGTGCCTTATGTAATTCCGGCGAGCAATCACACtt                                                                                                           <  1:370023/301‑1 (MQ=255)`  
                                                                                                                                                                                                   `aaaaTAAGGGTCGAGCACACAAGGGGTATAAACTTTCAGACGAAGACGTAATGGCAATCATGGAGTCCAGCGAGTCCAATGTATCCTTAGCTCGCACCTATGG‑TGTCTCCCAACAGACTATTTGTGATATACGCAAAGGTAGGCGACATGGCAGGTTACGGCGCTAAAGGAATCCGAAAGGTTGGAGCGTTTCGCTCTGGCCTAGAGGACAAGGTTTCAAAGCAGTTGGAATCAAAAGGTATTAAATTCGAGTATGAAGAGTGTAAAGTGCCTTATGTAATTCCGGCGAGCAATCACACtt                                                                                                           <  1:469862/300‑1 (MQ=255)`  
                                                                                                                                                                                                    `tataagagacagCTCAAACAAGGGGTATAAACTTTCAGACGAAGACGTAATGGCAATCATGGAGTCCAGCGAGTCCAATGTATCCTTAGCTCGCACCTATGG‑‑GTCTCCAAACAGACTATTTGTGATATACGCAAAGGGAGGCGACATGGCAGGTTACGGCGCTAAAGGAATCCGAAAGGTTGGAGCGGTTCGCTCTGGCCTAGAGGACAAGGTTTCAAAGCAGTTGGAATCAAAAGGTATTAAATTCGAGTATGAAGAGTGGAAAGTGCCTTATGTAATTCCGGCGAGCAATCACACTTac                                                                                                         <  1:544882/290‑1 (MQ=255)`  
                                                                                                                                                                                                    `aaaTAAGGGTCGCTCACACAAGGGGTATAAACTTTCAGACGAAGACGTAATGGCAATCATGGAGTCCAGCGAGTCCAATGTATCCTTAGCTCGCACCTATGG‑TGTCTCCCAACAGACTATTTGTGATATACGCAAAGGGAGGCGACATGGCAGGTTACGGCGCTAAAGGAATCCGAAAGGTTGGAGCGTTTCGCTCTGGCCTAGAGGACAAGGTTTCAAAGCAGTTGGAATCAAAAGGTATTAAATTCGAGTATGACGAGTGGAAAGTGCCTTATGTAATTCCGGCGAGCAATCACACTTa                                                                                                          >  1:631768/1‑301 (MQ=255)`  
                                                                                                                                                                                                       `tAAGGGTCGCTCACACAAGGGGTATAAACTTTCAGACGAAGACGTAATGGCAATCATGGAGTCCAGCGAGTCCAATGTATCCTTAGCTCGCACCTATGG‑TGTCTCCCAACAGACTATTTGTGATATACGCAAAGGGAGGCGACATGGCAGGTTACGGCGCTAAAGGAATCCGAAAGGTTGGAGCGTTTCGCTCTGGCCTAGAGGACAAGGTTTCAAAGCAGTTGGAATCAAAAGGTATTAAATTCGAATATGAAGAGTGGAAAGTGCCTTATGTAATTCCGGCGAGCAATCACACTTacac                                                                                                       >  1:323983/1‑301 (MQ=255)`  
                                                                                                                                                                                                       `aaaGGGTCGCTCACACAAGGGGTATAAACTTTCAGACGAAGACGTAATGGCAATCATGGAGTCCAGCGAGTCCAATGTATCCTTAGCTCGCACCTATGG‑TGTCTCCCAACAGACTATTTGTGATATACGCAAAGGGAGGCGACATGGCAGGTTACGGCGCTAAAGGAATCCGAAAGGTTGGAGCGTTTCGCTCTGGCCTAGAGGACAAGGTTTCAAAGCAGTTGGAATCAAAAGGTATTAAATTCGAGTATGAAGAGTGGAAAGTGCCTTATGTAATTCCGGCGAGCAATCACACTTacac                                                                                                       <  1:239393/300‑1 (MQ=255)`  
                                                                                                                                                                                                       `aaaGGGTCGCTCAACCAAGGGGTATAAACTTTCAGCCGAAGACGTAATGGCAATCATGGAGTCCAGCGAGTCCAATGTATCCTTAGCTCGCACCTATGG‑TGTCTCCCAACAGACTATTTGTGATATACGCAAAGGGAGGCGACATGGCAGGTTACGGCGCTAAAGGAATCCGAAAGGTTGGAGCGTTTCGCTCTGGCCTAGAGGACAAGGTTTCAAAGCAGTTGGAATCAAAAGGTATTAAATTCGAGTATGAAGAGTGGAAAGTGCCTTATGTAATTCCGGCGAGCAATCACACTTacac                                                                                                       <  1:336641/300‑1 (MQ=255)`  
                                                                                                                                                                                                       `aaaGGGGCGCTCACACAAGGGGTATAAACTTTCAGACGAAGACGTAATGGCAATCATGGAGTCCAGCGAGTCCAATGTATCCTTAGCTCGCACCTATGG‑TGTCTCCCAACAGACTATTTGTTATATACGCAAAGGGAGGCGACATGGCAGGTTACGGCGCTAAAGGAATCCGAAAGGTTGGAGCGTTTCGCTCTGGCCTAGAGGACAAGGTTTCAAAGCAGTTGGAATCAAAAGGTATTAAATTCGAGTATGAAGAGTGGAAAGTGCCTTATGTAATTCCGGCGAGCAATCACACTTacac                                                                                                       <  1:82953/300‑1 (MQ=255)`  
                                                                                                                                                                                                               `gCTCACACAAGGGGTATAAACTTTCAGACGAAGACGTAATGGCAATCATGGAGTCCAGCGAGTCCAATGTATCCTTAGCTCGCACCTATGG‑TGTCTCCCAACAGACTATTTGTGATATACGCAAAGGGAGGCGACATGGCAGGTTACGGCGCTAAAGGAATCCGAAAGGTTGGAGCGTTTCGCTCTGGCCTAGAGGACAAGGTTTCAAAGCAGTTGGAATCAAAAGGTATTAAATTCGTGTATGAAGAGTGGAAAGTGCCTTATGTAATTCCGGCGAGCAATCACACTTacacctgtctct                                                                                               >  1:178867/1‑293 (MQ=255)`  
                                                                                                                                                                                                               `ccTCACACAAGGGGTATAAACTTTCAGACGAAGACGTAAGTGCAATCATGAAGTCCAGCGAGTCCAATGTATTCTTAGCTCGCACCTATGG‑TGTCTCCCAACAGACTATTTGTCATATACGCAAAGGGAGGCGACATGGCAGGTTACGGCGCTAAAGGAATCCGAAAGGTTGGAGCGTTTCGCTCTGTCCTAGAGGACAAGGTTTCAAAGCAGTTGGAATCAAAAGGTATTAAATTCGAGTATGAAGAGTGGAAAGTGCCTTATGTAATTCCGGCGAGCAATCACACTTACACTCCAGAct                                                                                               <  1:418034/300‑1 (MQ=255)`  
                                                                                                                                                                                                                    `cacaAGGGGTATAAACTTTCAGACGAAGACGTAATGGCAATCATGGAGTCCAGCGAGTCCAATGTATCCTTAGCTCGCACCTATGG‑TGTCTCCCAACAGACTATTTGTGATATACGCAAAGGGAGGCGACATGGCAGGTTACGGCGCTAAAGGAATCCGAAAGGTTGGAGCGTTTCGCTCTGGCCTAGAGGTCAAGGTTTCAAAGCAGTTGGAATCAAAAGGTATTAAATTCGAGTATGAAGAGTGGAAAGTGCCTTATGTAATTCCGGCGAGCAATCACACTTacacctgtctcttatac                                                                                          >  1:431466/1‑288 (MQ=255)`  
                                                                                                                                                                                                                    `cacaAGGGGTATAAACTTTCAGACGAAGACGTAATGGCAATCATGGAGTCCAGCGAGTCCAATGTATCCTTAGCTCGCACCTATGG‑TGTCTCCCAACAGACTATTTGTGATATACGCAAAGGGAGGCGACATGGCAGGTTACGGCGCTAAAGGAATCCGAAAGGTTGGAGCGTTTCGCTCTGGCCTAGAGGACAAGGTTTCAAAGCAGTTGGAATCAAAAGGTATTAAATTCGAGTATGAAGAGTGGAAAGTGCCTTATGTAATTCCGGCGAGCAATCACACTTACACTCCAGACTTCTTa                                                                                          >  1:555552/1‑301 (MQ=255)`  
                                                                                                                                                                                                                    `cacaAGGGGTATAAACTTTCAGACGAAGACGTAATGGCAATCATGGAGTCCAGCGAGTCCAATGTATCCTTAGCTCGCACCTATGG‑TGTCTCCCAACAGACTACTTGTGATATACGCAAAGGGAGGCGACATGGCAGGTTACGGCGCTAAAGGAATCCGAAAGGTTGGAGCGTTTCGCTCTGGCCTAGAGGACAAGGTTTCAAAGCAGTTGGAATCAAAAGGTATTAAATTCGAGTATGAAGAGTGGAAAGTGCCTTATGTAATTCCGGCGAGCAATCacacctgtctcttatacacatct                                                                                          >  1:381072/1‑282 (MQ=255)`  
                                                                                                                                                                                                                          `gggTATAAACTTTCAGACGAAGACGTAATGGCAATCATGGAGTCCAGCGAGTCCAATGTATCCTTAGCTCGCACCTATGG‑TGTCTCCCAACAGACTATTTGTGATATACGCAAAGGGAGGCGACATGGCAGGTTACGGCGCTAAAGGAATCCGAAAGGTTGGAGCGTTTCGCTCTGGCCTAGAGGACAAGGTTTCAAAGCAGTTGGAATCAAAAGGTATTAAATTCGAGTATGAAGAGTGGAAAGTGCCTTATGTAATTCCGGCGAGCAATCACACTTACACTCCAGACTTCTTACTTCCa                                                                                    >  1:272140/1‑301 (MQ=255)`  
                                                                                                                                                                                                                            `gTATAAACTTTCAGACGAAGACGTAATGGCAATCATGGAGTCCAGCGAGTCCAATGTATCCTTAGCTCGCACCTATGG‑TGTCTCCCAACAGACTATTTGTGATATACGCAAAGGGAGGCGACATGGCAGGTTACGGCGCTAAAGGAATCCGAAAGGTTGGAGCGTTTCGCTCTGGCCTAGAGGACAAGGTTTCAAAGCAGTTGGAATCAAAAGGTATTAAATTCGAGTATGAAGAGTGGAAAGTGCCTTATGTAATTCCGGCGAGCAATCACACTTACACTCCAGACTTCTTACTTCCaaa                                                                                  >  1:170887/1‑301 (MQ=255)`  
                                                                                                                                                                                                                                     `ttCAGACGAAGACGTAATGGCAATCATGGAGTCCAGCGAGTCCAATGTATCCTTAGCTCGCACCTATGG‑TGTCTCCCAACAGACTATTTGTGATATACGCAAAGGGAGGCGACATGGCAGGTTACGGCGCTAAAGGAATCCGAAAGGTTGGAGCGTTTCGCTCTGGCCTAGAGGACAAGGTTTCAAAGCAGTTGGAATCAAAAGGTATTAAATTCGAGTATGAAGAGTGGAAAGTGCCTTATGTAATTCCGGCGAGCAATCACACTTACACTCCAGACTTCTTACTTCCAAACGGTAGAtt                                                                         <  1:607579/301‑1 (MQ=255)`  
                                                                                                                                                                                                                                                     `aTGGCAATCATGGAGTCCAGCGAGTCCAATGTATCCTTAGCTCGCACCTATGG‑TGTCTCCCAACAGACTATTTGTGATATACGCAAAGGGAGGCGACATGGCAGGTTACGGCGCTAAAGGAATCCGAAAGGTTGGAGCGTTTCGCTCTGGCCTAGAGGACAAGGTTTCAAAGCAGTTGGAATCAAAAGGTATTAAATTCGAGTATGAAGAGTGGAAAGTGCCTTATGTAATTCCGGCGAGCAATCACACTTACACTCCAGACTTCTTACTTCCAAACGGTATATTCGTTGAGACAAAGGGt                                                         >  1:65981/1‑301 (MQ=255)`  
                                                                                                                                                                                                                                                      `tGGTAATCAGGGAGCCCAGCGAGTCCAATGTATCCTTAGCTCGCACCTATGG‑TGTCTCCCAAAAGACTATTTGTGATATACGCAAAGGGAGGCGACATGGCAGGTTACGGCGCTAAAGGAATCCGAAAGGTTGGAGCGTTTCGCTCTGGCCTAGAGGACAAGGTTTCAAAGCAGTTGGAATCAAAAGGTATTAAATTCGAGTATGAAGAGTGGAAAGTGCCTTATGTAATTCCGGCGAGCAATCACACTTACACTCCAGACTTCTTACTTCCAAACGGTATATTCGTTGAGACAAAGGGTc                                                        <  1:44714/301‑1 (MQ=255)`  
                                                                                                                                                                                                                                                              `aatgtgtttaagagacagaaTGTATCCTTAGCTCGCACCTATGG‑TGTCTCCCAACAGACTATTTGTGATATACGCAAAGGGAGGCGACATGGCAGGTTACGGCGCTAAAGGAATCCGAAAGGTTGGAGCGTTTCGCTCTGGCCTAGAGGACAAGGTTTCAAAGCAGTTGGAATCAAAAGGTATTAAATTCGAGTATGAAGAGTGGAAAGTGCCTTATGTAATTCCGGCGAGCAATCACACTTACACTCCAGACTTCTTACTTCCAAACGGTATATTCGTTGAGACAAAGGGTCTGTGGGaa                                                <  1:300899/283‑1 (MQ=255)`  
                                                                                                                                                                                                                                                                  `ggagatgtgtataagagacagccTTAGCTCGCACCTATGG‑TGTCTCCCAACATACTATTTGTGATATACGCAAAGGGAGGCGACATGGCAGGTTACGGCGCTAAAGGAATCCGAAAGGTTGGAGCGTTTCGCTCTGGCCTAGAGGACAAGGTTTCAAAGCAGTTGTAATCAAAAGGTATTAAATTCGAGTATGAAGAGTGGAAAGTGCCTTATGTAATTCCGGCGAGCAATCACACTTACACTCCAGACTTCTTACTTCCAAACGGTATATTCGTTGAGACAAAGGGTCTGTGGGAAAGCg                                            <  1:202039/280‑1 (MQ=255)`  
                                                                                                                                                                                                                                                                         `ctcggagatgtgtataagagacagCACCTATGG‑TGTCTCCCAACAGACTATTTGTGATATACGCAAAGGGAGGCGACATGGCAGGTTACGGCGCTAAAGGAATCCGAAAGGTTGGAGCGTTTCGCTCTGGCCTAGAGGACAAGGTTTCAAAGCAGTTGGAATCAAAAGGTATTAAATTCGAGTATGAAGAGTGGAAAGTGCCTTATGTAATTCCGGCGAGCAATCACACTTACACTCCAGACTTCTTACTTCCAAACGGTATATTCGTTGAGACAAAGGGTCTGTGGGAAAGCGATGATag                                     <  1:151474/278‑1 (MQ=255)`  
                                                                                                                                                                                                                                                                            `gTCCAATGTATCCTTAGCTCGCACCTATGG‑TGTCTCCCAACAGACTATTTGTGATATACGCAAAGGGAGGCGACATGGCAGGTTACGGCGCTAAAGGAATCCGAAAGGTTGGATCGTTTCGCTCTGGCCTAGAGGACAAGGTTTCAAAGCAGTTGGAATCAAAAGGTATTAAATTCGAGTATGAAGAGTGGAAAGTGCCTTATGTAATTCCGGCGAGCAATCACACTTACACTCCAGACTTCTTACTTCCAAACGGTATATTCGTTTATACAAAGGGTCTGTggctgtctcttatacaaat                                  >  1:55021/1‑284 (MQ=255)`  
                                                                                                                                                                                                                                                                                 `aTGTATTCTTAGCTCGCACCTATGG‑TGTCTCCCAACAGACTAGTGGTGAGAGACTCAAAGGGTGGCGAAATGGCAGGTTACGGGGCTAAAGGAATCCAAAAGGTTCGAACGTTTCGCTCTGGCCTAGAAGACAAGCTTTTAAAGCAATTGGAATAAAAAGGTATTAAATTCGAGGATGAAGAGTGGAAAGTGCCTTATGAAATGACGGCGAGCAATCACACTTACACTCCAGACTTCTTACTTCCAAACGGTATATTCGTTGAGACAAAGGGTCTGTGGGAAAGCGATGATAGAAAGAAGc                             <  1:402178/301‑1 (MQ=255)`  
                                                                                                                                                                                                                                                                                 `aTGTATCCTTAGCTCGCACCTATGG‑TGTCTCCCAACAGACTATTTGTGATATACGCAAAGGGAGGCGACATGGCAGGTTACGGCGCTAAAGGAATCCGAAAGGTTGGAGCGTTTCGCTCTGGCCTAGAGGACAAGGTTTCAAAGCAGTTGGAATCAAAAGGTATTAAATTCGAGTATGAAGAGTGGAAAGTGCCTTATGTAATTCCGGCGAGCAATCACACTTACACTCCAGACTTCTTACTTCCAAACGGTATATTCGTTGATACAAAGGGTCTGTGGGAAAGCGATGATAGAAAGAAGc                             >  1:642384/1‑301 (MQ=255)`  
                                                                                                                                                                                                                                                                                 `aTGTATCCTTAGCTCGCACCTATGG‑TGTCTCCCAACAGACTATTTGTGATATACGCAAAGGGAGGCGACATGGCAGGTTACGGCGCTAAAGGAATCCGAAAGGTTGGAGCGTTTCGCTCTGGCCTAGAGGACAAGGTTTCAAAGCAGTTGGAATCAAAAGGTATTAAATTCGAGTATGAAGAGTGGAAAGTGCCTTATGTAATTCCGGCGAGCAATCACACTTACACTCCAGACTTCTTACTTCCAAACGGTATATTCGTTGAGACAAAGGGTCTGTGGGAAAGCGATGATAGAAAGAAGc                             >  1:600989/1‑301 (MQ=255)`  
                                                                                                                                                                                                                                                                                   `tgtataagagacagCAACTATGG‑TGTCTCCCAACAGACTATTTGTGATATACGCAAAGGGAGGCGACATGGCAGGTTACGGCGCTAAAGGAATCCGAAAGGTTGGAGCGTTTCGCTCTGGCCTAGAGGACAAGGTTTCAAAGCAGTTGGAATCAAAAGGTATTAAATTCGAGTATGAAGAGTGGAAAGTGCCTTATGTAATTCCGGCGAGCAATCACACTTACACTCCAGACTTCTTACTTCCAAACGGTATATTCGTTGAGACAAAGGGTCTGTGGGAAAGCGATGATAGAAAGAAGCAc                           <  1:527523/288‑1 (MQ=255)`  
                                                                                                                                                                                                                                                                                      `tCCTTAGCTCGCACCTATGG‑TGTCTCCCAACAGACTATTTGTGATATACGCAAAGGGAGGCGACATGGCAGGTTACGGCGCTAAAGGAATCCGAAAGGTTGGAGCGTTTCGCTCTGGCCTAGAGGACAAGGTTTCAAGGCAGTTGGAATCAAAAGGTATTAAATTCGAGTATGAAGAGTGGAAAGTGCCTTATGTAATTCCGGCGAGCAATCACACTTACACTCCAGACTTCTTACTTCCAAACGGTATATTCGTTGAGACAAAGGGTCTGTGGGAAAGCGATGATAGAAAGAAGCACttt                        >  1:288222/1‑300 (MQ=255)`  
                                                                                                                                                                                                                                                                                      `tCCTTAGCTCGCACCTATGG‑TGTCTCCCAACAGACTATTTGTGATATACGCAAAGGGAGGCGACATGGCAGGTTACGGCGCTAAAGGAATCCGAAAGGTTGGAGCGTTTCGCTCTGGCCTAGAGGACAAGGTTTCAAAGCAGTTGGAATCAAAAGGTATTAAATTCGAGTATGAAGAGTGGAAAGTGCCTTATGTAATTCCGGCGAGCAATCACACTTACACTCCAGACTTCTTACGTCCAACCGGTATATTCGTTGAGACCAAGGGTCTGTGGGAAAGCGATGATAGAAAGAAGCACttt                        >  1:364623/1‑300 (MQ=255)`  
                                                                                                                                                                                                                                                                                      `tCCTTAGCTCGCACCCATGG‑TGTCTCCCATCAGACTATTTGTTATAGACGCAAAGGTAGGCGACAGGGCTAGTTAAGGCGCTAAAGGAATCTGACAGGTTGGAGCTTTTCGCTCTGGTCTAGTGGACAAGGTTACAAATCCTTTGGAATCAAAAGGTATTAAATTCTAGTATGAAGAGTGGAAAGTTCCTTATGTAATTCCGGCGAGCAATCACACTTACACTCCAGACTTCTTACTTCCAAACGGTATATTCGTTGAGACAAAGGGTCTGTGGGAAAGCGATGATAGAAAGAAGCACtta                        <  1:277597/301‑1 (MQ=255)`  
                                                                                                                                                                                                                                                                                           `aGCTCGCACCTATGG‑TTTCTCCCAACAGACTATTTGTGATATACGCAAAGGGAGGCGAAATGGCAGGTTACGGCGCTAAAGGAATCCGAAAGGTTGGAGCGTTTCGCTCTGGCCTAGAGGACAAGGTTTCAAAGCAGTTGGAATCAAAAGGTATTAAATTCGAGTATGAAGAGTGGAAAGTGCCTTATGTAATTCCGGCGAGCAATCACACTTACACTCCAGACTTCTTACTTCCAAACGGTATATTCGTTGAGACAAAGGGTCTGTGGGAAAGCGATGATAGAAAGAAGCACTTattaat                   <  1:464180/301‑1 (MQ=255)`  
                                                                                                                                                                                                                                                                                           `aGCTCGCACCTATGG‑TGTCTCCCAACAGACTATTTGTGATATACGCAAAGGGAGGCGACATGGCAGGTTACGGCGCTAAAGGAATCCGAAATGTTGGAGCGTTTCGCTCTGGCCTAGAGGACAAGGTTTCAAAGCAGTTGGAATCAAAAGGTATTAAATTCGAGTATGAAGAGTGGAAAGTGCCTTATGTAATTCCGGCGAGCAATCACACTTACACTCCAGACTTCTTACTTCCAAACGGTATATTCGTTGAGACAAAGGGTCTGTGGGAAAGCGATGATAGAAAGAAGCACTTattaat                   >  1:293959/1‑301 (MQ=255)`  
                                                                                                                                                                                                                                                                                           `aGCTCGCACCTATGG‑TGTCGTCCAACAGAATAGTTGGGATATACGCAAAGGGAGGCGACATGGCAGGTTACGGCGCTAAAGGAATCCGAAAGGTTGGAGCGTTTCGCTCTGGCCTAGAGGACAAGGTTTCAAAGCAGTTGGAATCAAAAGGTATTAAATTCGAGTATGAAGAGTGGAAAGTGCCTTATGTAATTCCGGCAAGCAATCACACTTACACTCCAGACTTCTTACTTCCAAACGGTATATTCGTTGAGACAAAGGGTCTGTGGGAAAGCGATGATAGAAAGAAGCACTTattaat                   <  1:77124/301‑1 (MQ=255)`  
                                                                                                                                                                                                                                                                                           `aGCACGAACCTATGG‑TGTATCCCAGAAGACTATTTGTGATATACGCAAAGGGAGGCGACATGGCAGGTTACGGCGCTAAAGGAATCCGAAAGGTTGGAGCGTTTCGCTCTGGCCTAGAGGACAAGGTTTCAAAGCAGTTGGAATCAAAAGTTATTAAATTCGAGTATGAAGAGTGGAAAGTGCCTTATGTAATTCCGGCGAGCAATCACACTTACACTCCAGAATTCTTACTTCCAAACGGTATATTCGTTGAGACAAAGGGTCTGTGGGAAAGCGATGATAGAAAGAAGCACTTattaat                   <  1:637150/301‑1 (MQ=255)`  
                                                                                                                                                                                                                                                                                            `gCTCGCACCTATGG‑TGTCTCCCAACAGACTATTTGTGATATACGCAAAGGGAGGCGACATGGCAGGTTACGGCGCTAAAGGAATCCGAAAGGTTGGAGCGTTTCGCTCTGGCCTAGAGGACAAGGTTTCAAAGCAGTTGGAATCAAAAGGTATTAAATTCGAGTATGAAGAGTGGAAAGTGCCTTATGTAATTCCGGCGAGCAATCACACTTACACTCCAGACTTCTTACTTCCAAACGGTATATTCGTTGAGACAAAGGGTCTGTGGGAAAGCGATGATAGAAAGAAGCACTTattaatt                  >  1:666247/1‑301 (MQ=255)`  
                                                                                                                                                                                                                                                                                              `tagaaacTATGG‑TTGCTCCCAACAGAATATGTGTGATATACGCAAAGGTAGGCGACATGGCAGGTTACGGCGCTAAAGGAATCCGAAAGGTTGGAGCGTTTCGCTCTGGCCTAGAGGACAAGGTTTCAAAGCAGTTGGAATCAAAAGGTATTAAATTCGAGTATGAAGAGTGGAAAGTGCCTTATGTAATTCCGGCGAGCAATCACACTTACACTCCAGACTTCTTACTTCCAAACGGTATATTCGTTGAGACAAAGGGTCTGTGGGAAAGCGATGATAGAAAGAAGCACTTATTAATTAg                <  1:128083/295‑1 (MQ=255)`  
                                                                                                                                                                                                                                                                                                `ccACCTATGG‑TGTCTCCCAACAGACTATTTGTGATATACGCAAAGGGAGGCGACATGGCAGGTTACGGCGCTAAAGGAATCCGAAAGGTTGGAGCGTTTCGCTCTGGCCTAGAGGACAAGGTTTCAAAGCAGTTGGAATCAAAAGGTATTAAATTCGAGTATGAAGAGTGGAAAGTTCCTTATGTAATTCCGGCGAGCAATCACACTTACACTCCAGACTTCTTACTTCCAAACGGTATATTCGTTGAGACAAAGGGTCTGTGGGAAAGCGATGATAGAAAGAAGCACTTATTAATTAggg              <  1:10670/300‑1 (MQ=255)`  
                                                                                                                                                                                                                                                                                                   `ccTATGG‑TGTCTCCCAACAGACTATTTGTGATATACGCAAAGGGAGGCGACATGGCAGGTTACGGCGCTAAAGGAATCCGAAAGGTTGGAGCGTTTCGCTCTGGCCTAGAGGACAAGGTTTCAAAACAGTTGGAATCAAAAGGTATTAAATTCGAGTATGCAGAGTGGAAAGTGCCTTATGTAATTCCGGCGAGCAATCACACTTACTCTCCAGACTTCTTACTTTCAAACGGTATATTCGTTGAGACAAAGGGTCTGTTGGAAAGCGATGATAGAAAGAATCACCTATTAATTAgggctg           >  1:395166/1‑298 (MQ=255)`  
                                                                                                                                                                                                                                                                                                      `aTGG‑TGTCTCCCAACAGACTATTTGTGATATACGCAAAGGGAGGCGACATGGCAGGTTACGGCGCTAAAGGAATCCGAAAGGTTGGAGCGTTTCGCTCTGGCCTAGAGGACAAGGTTTCAAAGCAGTTGGAATCAAAAGGTATTAAATTCGAGTATGAAGAGTGGAAAGTGCCTTATGTAATTCCGGCGAGCAATCACACTTACACTCCAGACTTCTTACTTCCAAACGGTATATTCGTTGAGACAAAGGGTCTGTGGGAAAGCGATGATAGAAAGAAGCACTTATTAATTAGGGagcagc        <  1:614496/301‑1 (MQ=255)`  
                                                                                                                                                                                                                                                                                                        `gg‑TGTCTCCCAACCGACGAATTGTGATATACGCAAAGGGAGGCGACATGGCAGGTTACGGCGCTAAAGGAATACGAAAGGTTGGAGCGTTTCGCTCTGGCCTAGAGGACAAGGTTTCAAAGCAGTTGGAATCAAAAGGTATTAAATTCGAGTATGAAGAGTGGAAAGTGCCTTATGTAATTCCGGCGAGCAATCACACTTACACTCCAGACTTCTTACTTCCAAACGGTATATTCGTTGAGACAAAGGGTCTGTGGGAAAGCGATGATAGAAAGAAGCACTTATTAATTAGGGAGCAGCAc      <  1:121561/301‑1 (MQ=255)`  
                                                                                                                                                                                                                                                                                                         `g‑tgtCTCCCAACAGACTATTTGTGATATACGCAAAGGGAGGCGACATGGCAGGTTACGGCGCTAAAGGAATCCGAAAGGTTGGAGCGTTTCGCTCTGGCCTAGAGGACAAGGTTTCAAAGCAGTTGGAATCAAAAGGTATTAAATTCGAGTATGAAGAGTGGAAAGTGCCTTATGTAATTCCGGCGAGCAATCACACTTACACTCCAGACTTCTTACTTCCAAACGGTATATTAGTTGAGACAAAGGGTCTGTGGGAAAGCGATGATAGAAAGAAGCACTTATTAATTAGGGAGCAGCAcc     >  1:206493/1‑301 (MQ=255)`  
                                                                                                                                                                                                                                                                                                           `tgtCTCCCAACAGACTAATTGTGATATACGCAAAGGGAGGCGACATGGCAGGTTACGGCGCTAAAGGAATCCGAAAGGTTGGAGCGTTTCGCTCTGGCCTAGAGGACAAGGTTTCAAAGCAGTTGGAATCAAAAGGTATTAAATTCGAGTATGAAGAGTGGAAAGTGCCTTATGTAATTCCGGCGAGCAATCACACTTACACTCCAGACTTCTTACTTCCAAATGGTATATTCGTTGAGACAAAGGGTCTGTGGGAAAGCGATGATAGAAAGAAGCACTTATTAATTAGGGAGCAGCAccc    <  1:442289/301‑1 (MQ=255)`  
                                                                                                                                                                                                                                                                                                            `gtCTCCCAACAGACTATTTGTGATATACGCAAAGGGAGGCGACATGGCAGGTTACGGAGCTAAAGGAATCCGAAAGGTTGGAGCGTTTCGCTCTGGCCTAGAGGACAAGGTTTCAAAGCAGTTGGAATCAAAAGGTATTAAATTCGAGTATGAAGAGTGGAAAGTGCCTTATGTAATTCCGGCGAGCAATCACACTTACACTCCAGACTTCTTACTTCCAAACGGTATATTCGTTGAGACAAAGGGTCTGTGGGCAAGCGATGATAGACAGACGCACttattctgtctcttatacacatct   >  1:2534/1‑282 (MQ=255)`  
                                                                                                                                                                                                                                                                                                             `tctcCCAACAGACTATTTGTGATATACGCAAAGGGAGGCGACATGGCAGGTTACGGCGCTAAAGGAATCCGAAAGGTTGGAGCGTTTCGCTCTGGCCTAGAGGACAAGGTTTCAAAGCAGTTGGAATCAAAAGGTATTAAATTCGAGTATGAAGAGTGGAAAGTGCCTTATGTAATTCCGGCGAGCAATCACACTTACACTCCAGACTTCTTACTTCCAAACGGTATATTCGTTGAGACAAAGGGTCTGTGGGAAAGCGATGATAGAAAGAAGCACTTATTAATTAGGGAGCAGCACCCCg  >  1:561281/1‑301 (MQ=255)`  
                                                                                                                                                                                                                                                                                                             `tctcCCAACAGACTATTTGTGATATACGCAAAGGGAGGCGACATGGCAGGTTACGGCGCTAAAGGAATCCGAAAGGTTGGAGCGTTTCGCTCTGGCCTAGAGGACAAGGTTTCAAAGCAGTTGGAATCAAAAGGTATTAAATTCGAGTATGAAGAGTGGAAAGTGCCTTATGTAATTCCGGCGAGCAATCACACTTACACTCCAGACTTCTTACTTCCAAACGGTATATTCGTTGAGACAAAGGGTCTGTGGGAAAGCAATGATAGAAAGAAGCACTTATTAATTAGGGAGCAGCACCCCg  >  1:534362/1‑301 (MQ=255)`  

                                                                                                                                                                                                                                                                                                             `|`                                                                                                                                                                                                                                                                                                              
`AGTGGCAGGGCGCTACGAACAATAAAGGGTACGGGCAGGTGTGGTGCAGCAATACCGGAAAGGTTGTCTACTGTCATCGCGTAATGTCTAATGCTCCGAAAGGTTCTACCGTCCTGCACTCCTGTGATAATCCATTATGTTGTAACCCTGAACACCTATCCATAGGAACTCCAAAAGAGAACTCCACTGACATGGTAAATAAGGGTCGCTCACACAAGGGGTATAAACTTTCAGACGAAGACGTAATGGCAATCATGGAGTCCAGCGAGTCCAATGTATCCTTAGCTCGCACCTATGG‑TGTCTCCCAACAGACTATTTGTGATATACGCAAAGGGAGGCGACATGGCAGGTTACGGCGCTAAAGGAATCCGAAAGGTTGGAGCGTTTCGCTCTGGCCTAGAGGACAAGGTTTCAAAGCAGTTGGAATCAAAAGGTATTAAATTCGAGTATGAAGAGTGGAAAGTGCCTTATGTAATTCCGGCGAGCAATCACACTTACACTCCAGACTTCTTACTTCCAAACGGTATATTCGTTGAGACAAAGGGTCTGTGGGAAAGCGATGATAGAAAGAAGCACTTATTAATTAGGGAGCAGCACCCCG  >  NC_001604.1/9915‑10515`

  

Alignment Legend

Aligned base mismatch/match (shaded by quality score): `ATCG/ATCG` `< 3 ≤` `ATCG/ATCG` `< 11 ≤` `ATCG/ATCG` `< 22 ≤` `ATCG/ATCG` `< 34 ≤` `ATCG/ATCG` `< 38 ≤` `ATCG/ATCG`

Unaligned base: `atcg`    Masked matching base: `atcg`    Alignment gap: `‑`    Deleted base: `‑`

Reads not counted as support for junction

read\_name Not counted due to insufficient overlap past the breakpoint.

read\_name Not counted due to not crossing MOB target site duplication.
